# Comparison of Virtual Reality Exergames and Nature Videos on Attentional Performance: A Single-Session Study

**DOI:** 10.3390/brainsci14100972

**Published:** 2024-09-26

**Authors:** Elena Rodríguez-Rodríguez, Joaquín Castillo-Escamilla, Francisco Nieto-Escamez

**Affiliations:** 1 Department Psychology, University of Almeria, La Cañada de San Urbano, 04120 Almeria, Spain; psiconeurovr@gmail.com (E.R.-R.); jce450@ual.es (J.C.-E.); 2 Research Center for Wellbeing and Social Inclusion (CIBIS), University of Almeria, La Cañada de San Urbano, 04120 Almeria, Spain

**Keywords:** virtual reality (VR), attentional blink (AB), flanker task, cognitive training, exergames, nature exposure

## Abstract

Background/Objectives: This study aimed to investigate the acute effects of a single session of a VR exergame (*Beat Saber*) and a VR nature video (Ireland 4K) on attentional performance, using the Flanker and Attentional Blink (AB) tasks. The objective was to assess whether these VR interventions could enhance attentional control, as measured by improvements in response times and accuracy. Methods: A total of 39 psychology students, aged 19–25, were randomly assigned to one of three groups: VR exergame, VR nature video, or control. Participants completed the Flanker and AB tasks before and after the intervention. A repeated measures design was employed to analyze changes in response times and accuracy across pre- and post-test sessions. Results: The study revealed significant improvements in response times and accuracy across all groups in the post-test measures, indicating a strong training effect. In the AB task, shorter stimulus onset asynchrony (SOA) led to decreased accuracy and slower response times, emphasizing the difficulty in processing closely spaced targets. The interaction between Type and Group in response times for target stimuli suggested that the intervention types differentially influenced processing speed in specific conditions. Conclusions: The findings suggest that while brief VR interventions did not produce significant differences between groups, the training effect observed highlights the influence of task-specific factors such as SOA and target presence. Further research is needed to explore whether longer or repeated VR sessions, as well as the optimization of task-specific parameters, might lead to more pronounced cognitive benefits.

## 1. Introduction

In recent years, advances in technology have enabled the development of new tools for cognitive training, most notably video games and immersive virtual reality (VR). These technologies not only offer new forms of entertainment but also provide opportunities to improve cognitive functions, particularly attentional functions [1,2]. VR has established itself as an innovative tool in cognitive research, especially in the study of attention. Its ability to create immersive and controlled environments allows researchers to explore various facets of human behavior in conditions that simulate real experiences, facilitating experimental manipulation and the collection of accurate data [3], which enhances its effectiveness in neuropsychological rehabilitation, especially in attention training [4].

From a neuroanatomical perspective, fMRI studies show that VR experiences can activate regions associated with attentional processes, such as the dorsal attentional network (DAN), which is crucial for top-down attentional control, and the V3A region of the visual cortex, which would imply an enhancement in attentional engagement compared to traditional methods by creating more realistic visual environments [5]. This type of engagement is crucial for attention restoration and is particularly valuable in populations with attentional deficits, as seen in neurological conditions.

Furthermore, VR interventions, especially those combined with physical activity (exergames), are increasingly investigated for their role in enhancing cognitive functions, including attentional functions in healthy [6] and clinical populations [7,8]. This can be attributed to the interaction between motor and cognitive systems, which leads to improved attentional processing [5].

Exergames such as *Beat Saber* are particularly intriguing in this context due to their ability to engage players in tasks requiring high precision, visuomotor coordination, and rapid decision-making. The highly stimulating environment offered by these games could potentially strengthen skills such as selective and sustained attention, as well as information processing speed [9]. On the other hand, immersive VR experiences can be designed to create environments that provide a full sensory experience, influencing attentional abilities in a controlled environment [10].

Furthermore, exposure to relaxing virtual scenarios has also been shown to positively affect executive mental functioning, specifically in the restoration and recovery of attentional function [11]. Attentional restoration can enhance voluntary attention, which is often depleted by overuse. This is critical for improving performance in tasks requiring inhibitory control and selective attention [12]. Relaxing scenarios can also aid in cognitive recovery after performing tasks that demand high levels of attention, leading to better performance in subsequent tasks [13].

Overall, video games, especially those incorporating VR, have been shown to impact brain function and behavior, with gamers often excelling in various visuospatial and attention-related tasks. Among these tasks are the Flanker and Attentional Blink (AB) tasks [14]. The Flanker task measures the ability to focus attention on relevant stimuli while ignoring distractors, assessing selective attention, inhibitory control, and response accuracy [15]. Meanwhile, the AB task evaluates the ability to rapidly process information in a sequence of visual events by determining the time required to regain attention after a brief lapse [16].

Finally, studies employing multiple VR sessions demonstrate that extended engagement can lead to significant cognitive enhancements, particularly in attention, and suggest that sustained VR exposure creates cumulative cognitive benefits [17,18], making them the gold standard for assessing VR’s potential in cognitive training. However, the question remains whether short-term interventions, such as single VR sessions, can also offer meaningful improvements in attention. Understanding whether cognitive benefits can be detected after just one session is critical for developing scalable, accessible interventions that may be deployed in time-limited environments. Gao et al. [19] observed temporary improvements in concentration after playing 10 min of a casual exergame. And data from our laboratory (article in preparation) indicate that even a single session using *Beat Saber*, produced significant improvements in visual acuity. This suggests that short-term VR exposure may provide immediate cognitive benefits, at least in specific domains such as visual processing.

### Hypotheses

We hypothesize that exposure to the VR exergame *Beat Saber* and the VR nature experience Ireland 4K will produce differential enhancements in cognitive and attentional functions, as quantified by the AB and Flanker tasks. *Beat Saber*, characterized by its high level of engagement [20] and a combination of mental and physical activity [21], is expected to specifically improve rapid cognitive processing and executive functions. It has also been reported that *Beat Saber* training improved visual attention in children with reading difficulties [22], involving factors such as saccadic eye movements and visuospatial coordination. Another potential consequence of this type of training could be the reduction in the attentional blink effect by enhancing T2 detection accuracy during short intervals after T1, indicating a heightened ability to manage rapid attention shifts and multiple stimuli [21], crucial for tasks requiring quick cognitive responses [23,24]. Furthermore, *Beat Saber*’s influence on neural pathways related to perceptual and cognitive subprocesses may lead to decreased reaction times and lower error rates in the Flanker task, indicating improved conflict resolution abilities [25,26,27].

Conversely, the Ireland 4K nature scenario, known for its immersive and serene settings, is projected to enhance overall cognitive capacity, particularly in sustaining attention and reducing cognitive fatigue. This intervention might improve overall T2 detection rates across various temporal intervals in the AB task, reflecting a uniform enhancement of sustained attention due to its restorative effects on cognitive and emotional states [28]. For the Flanker task, while Ireland 4K may not drastically improve reaction times, it is expected to lead to more consistent and accurate responses across both congruent and incongruent trials, enhancing overall cognitive stability and reducing cognitive load [29].

## 2. Materials and Methods

### 2.1. Participants

The minimum sample size was calculated using G*Power 3.1.9.7 for three groups and 10 measurements, considering an effect size f of 0.25, an alpha error of 0.05, and a power of 0.95, yielding a required sample size of 27. The study included 39 participants (30 women and 9 men) aged 19 to 25 years (M = 21, SD = 1.99). All participants were psychology students from the University of Almeria. Participants were compensated with academic credit in exchange for their participation in the experiment. The sample was non-probabilistic, with participants randomly assigned to three groups: the VR Exergame Group (n = 14), the Nature Video Group (n = 13), and the Control Group (n = 12). Participants with any ocular, visual, or neurological pathology, or those following pharmacological treatments that might alter visual or attentional capacity, were excluded. Demographic data were collected during screening, and the study complied with Directive 2001/20/EC of the European Communities Council and the Declaration of Helsinki regarding research involving human subjects. Informed consent was obtained from all participants.

### 2.2. Materials

#### 2.2.1. Hardware

An HP laptop with a 15.6” full HD screen, 225 GB SSD, and 8 GB RAM was used for both tasks (HP, Palo Alto, CA, USA). VR exergame and nature video groups used Oculus Meta Quest 2 VR glasses (Meta Platforms, Menlo Park, CA, USA), featuring 128 GB storage, 1832 × 1920 resolution per eye, and 773 PPI, equipped with Fresnel lenses and two Touch controllers.

#### 2.2.2. Software

In terms of cognitive assessment software, we employed the Flanker task and the Attentional Blink task from the comprehensive PsyToolkit library [30,31]. These tasks can be downloaded from https://www.psytoolkit.org/experiment-library/flanker.html (accessed on 23 February 2024) [32] and https://www.psytoolkit.org/experiment-library/ab.html (accessed on 23 February 2024) [33], respectively.

##### Flanker Task

The Flanker task measures inhibitory control, response accuracy, and selective attention in the presence of two different types of stimuli [15]. For this study, we used a version of the Flanker task developed in the PsyToolkit software (https://www.psytoolkit.org/, accessed on 23 February 2024) [30], adapted from Duncan et al. [34]. The task involves the presentation of a sequence of five letters above a fixation point in the center of the screen. Participants are required to respond to the central letter, which serves as the target. They should press the ‘A’ key (located on the left) if the target letter is X or C or press the ‘L’ key (located on the right) if the target letter is V or B. The stimuli can be of two types: congruent (when the flankers require a similar response to the central letter) or incongruent (when the flankers require a different response than the central letter) [35] (Table 1).

**Figure 1:**
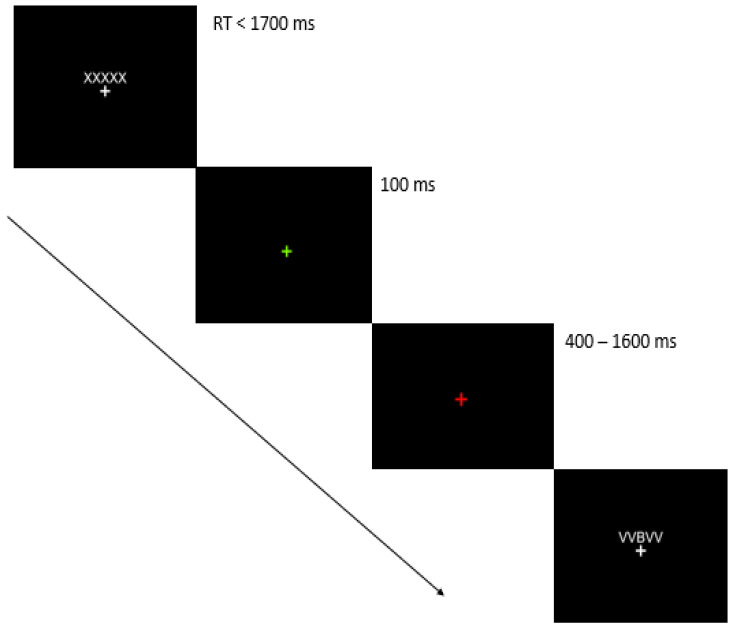
Stimuli and target conditions. Experimental process. Each stimulus sequence has a duration of 1700 ms. If the correct key is pressed, a green cross appears for 100 ms. If the wrong key is pressed, a red cross appears for 400 to 1600 ms. After the feedback, the next trial begins.

The task consists of 50 trials in total, with 25 congruent and 25 incongruent trials. After each trial, participants receive feedback indicating whether they pressed the key correctly or incorrectly. A green cross appears immediately after a correct response, while a red cross appears if the response was incorrect or if it took more than 1700 ms to respond (see Figure 1).

##### Attentional Blink (AB) Task

In the present study, two types of variables were recorded, namely accuracy and response times, each divided into congruent and incongruent conditions, resulting in a total of four variables. These were measured identically in both the pretest and post-test assessments. Better accuracy and lower response times indicate improved inhibitory control. Finally, the interference effect was measured by calculating the difference in response times between incongruent and congruent conditions, as this reflects the degree of difficulty introduced by the distractor flankers.

To assess temporal aspects of visual attention and detect rapidly presented sequential targets, we used the Attentional Blink (AB) task [16], based on the experiment by Duncan et al. [34]. In this task, participants detect and identify a target stimulus, which can appear as the first target (T1) or the second target (T2). Participants initiate the task by pressing the space key, followed by a semi-random delay of 0 to 500 ms. A blank screen with a fixation point and four surrounding boxes indicates the possible positions of the stimulus. In each trial, the target stimulus—a symbol in the shape of the letter L—may appear in T1 or T2 and is quickly followed by a masking pattern, or it may be absent altogether. The target stimulus is presented at different locations for each trial.

**Figure 2:**
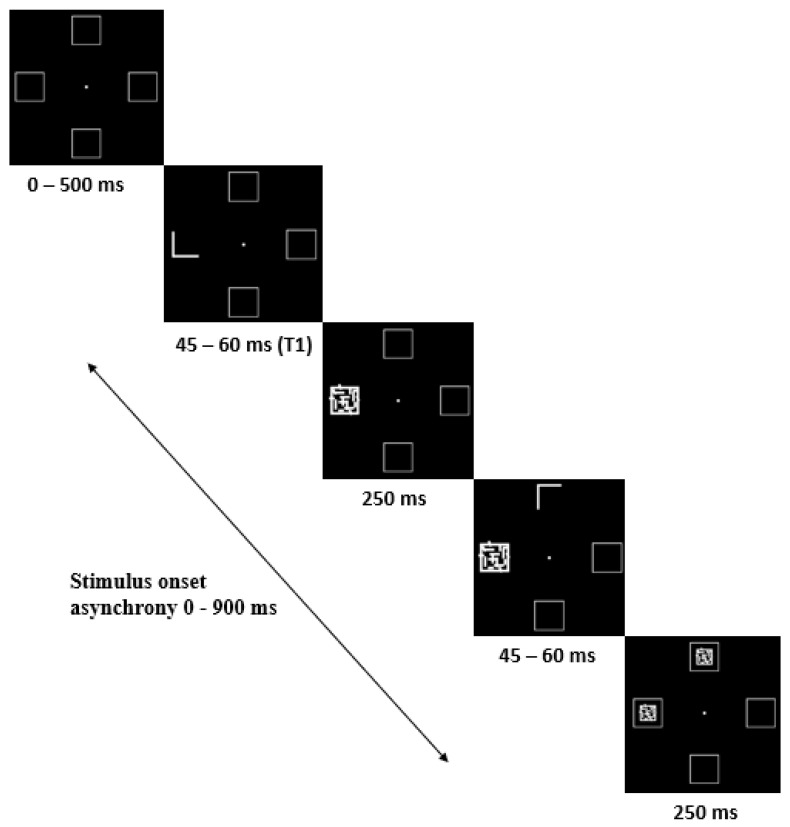
Stimulus sequence. Example trial where the target symbol in the shape of the letter ‘L’ is present as the first stimulus (T1).

Participants must press the B key if they have seen the target stimulus (regardless of whether it appeared as T1 or T2) or press the N key if they have not seen it. Feedback is provided after each trial to indicate whether the response was correct. The interval between T1 and T2 ranges from 0 to 900 ms, with stimuli measuring 0.5° × 0.6° and displayed for 45 to 60 ms, followed by a 250 ms masking pattern to limit visual persistence [34]. See Figure 2 for a sample trial. The task includes 100 trials: 50 with the stimuli present and 50 with the stimuli absent. A key feature of the AB task is the rapid succession of the first and second stimuli, as the second target often cannot be detected or identified when it appears shortly after the first [16]. Additionally, to measure how long a stimulus continues to occupy attentional capacity, targets may or may not be presented at randomly placed locations [36].

In this task, the two target stimuli do not appear together in the same trial. The percentage of correct answers is categorized into six possible conditions: type 1 (T1, SOA ≤ 300 ms); type 2 (T1, SOA > 300 ms); type 3 (T2, SOA ≤ 300 ms); type 4 (T2, SOA > 300 ms); type 5 null (neither T1 nor T2 appears, SOA > 300 ms); and type 6 null (neither T1 nor T2 appears, SOA ≤ 300 ms). This task measures cognitive flexibility and the ability to recover attention after temporal distractors, as well as the capacity to identify target stimuli following temporal interference. Since the Attentional Blink (AB) has been explained as the result of a structural limit on sequential information processing [37,38], this task serves as a measure of that limitation. The primary variable recorded in this task is the accuracy of responses after rapid serial visual processing.

##### Beat Saber

*Beat Saber* is a rhythm-based exergame (exercise and physical activity game) developed by Beat Games (Prague, Czech Republic) and released on 21 May 2019 [39,40]. The objective of the game is to slice a series of cubes to the beat of the music as they approach the player. Each cube is a different color (blue or red), and the player must use a correspondingly colored sword (blue or red) to slice the cube in the direction indicated by the arrow on the cube.

The challenge of the game lies in the high speed at which the cubes move, requiring players to concentrate intensely to identify the cube colors and the directions of the arrows. Players must remain highly attentive to both the cubes approaching from the front and those coming from behind due to the extreme speed at which they appear.

##### Ireland 4K

Ireland 4K is a YouTube video that features a variety of landscapes from Ireland, showcasing both aquatic and terrestrial scenery. In addition to the diverse visual content, the video is accompanied by relaxing music throughout its duration [41].

### 2.3. Procedure

The study was organized as follows: Participants were randomly allocated to one of three groups. After signing informed consent, the researcher provided the necessary instructions to all participants. Participants first completed the Flanker task and the Attentional Blink (AB) task sequentially. These tasks were administered before participants were assigned to their respective groups, which then received different interventions as described below.

For the VR exergame and nature video groups, participants used virtual reality goggles for approximately the first five minutes. With the help of the researcher, the goggles were adjusted to fit the participants’ head size and ensure proper settings for the intervention.

Participants in the VR exergame group engaged in a 20 min session of the game *Beat Saber*. All participants played the same song, “*Beat Saber*”, progressing through levels, starting with “Easy” and advancing to “Expert+” as they succeeded. The game instructions were narrated to participants during the setup phase.

Participants in the nature video group watched a 20 min segment of “Ireland 4K”, a one-hour film featuring natural landscapes, including coastal and mountain scenery, accompanied by relaxing music. The 4K Ireland video was displayed through the VR goggles in 2D screen mode.

Finally, participants in the control group spent 20 min in the laboratory, reading a series of magazines and flyers containing information about various activities at the University of Almeria. After the 20 min, they were not asked about the content they had read. Immediately following this period, the Flanker and AB tasks were administered again under the same conditions as before. The assessment tasks took between 15 and 20 min to complete (5 min for the Flanker task and 10–15 min for the Attentional Blink task), with the total duration of the experiment being one hour.

### 2.4. Study Design

The research design was a repeated measures experiment that included a pretest assessment, an intervention phase, and a post-test assessment. Data were analyzed using IBM SPSS Statistics 26 software, with the significance level set at *p* < 0.05. All data are presented as means (M), standard deviations (SD), and percentages (%). The Shapiro–Wilk test was used to assess the normality of the distribution, and the Levene test was used to assess homogeneity of variances. An initial Pearson correlation between the different variables of both tasks was conducted to check internal consistency and validity. The variables and the analyses performed are described below.

For the Flanker Task, the recorded variables were accuracy, response time (for both congruent and incongruent stimuli), and interference (the difference in response time between congruent and incongruent stimuli). The analysis of response times for congruent and incongruent stimuli was conducted using a 2 × 2 × 3 repeated measures ANOVA, with Group (VR VIDEO, VR EXERGAME, CONTROL) as a between-subjects factor, and Time (pre, post) and Type of stimuli (congruent, incongruent) as within-subjects factors. The interference effect was analyzed using a 2 × 3 repeated measures ANOVA, with Time (pre, post) as a within-subjects factor and Group (VR VIDEO, VR EXERGAME, CONTROL) as a between-subjects factor. Finally, total hits were analyzed using another 2 × 3 repeated measures ANOVA, and congruent and incongruent hits were analyzed using a 2 × 2 × 3 repeated measures ANOVA.

For the Attentional Blink (AB) task, several consecutive analyses were performed: A repeated measures ANOVA was conducted with Time (2 levels: pretest and post-test), Type (2 levels: present and non-present), and Group (3 levels: VR VIDEO, VR EXERGAME, CONTROL). Present and non-present trials were further divided into six variables: type 1 (T1, SOA < 300 ms), type 2 (T1, SOA > 300 ms), type 3 (T2, SOA < 300 ms), type 4 (T2, SOA > 300 ms), type 5 (NULL, SOA < 300 ms), and type 6 (NULL, SOA > 300 ms). The repeated measures ANOVA was then performed again using these criteria, with Type having six levels.

Two additional separate analyses were conducted to distinguish between present and non-present trials, as they differ in nature. One repeated measures ANOVA included Type with four levels (present trials: type 1 to type 4), and the other included Type with two levels (non-present trials: type 5 and type 6).

These analyses were performed for both accuracy and response times separately. Post hoc analyses were conducted using the Bonferroni correction, with a significance threshold set at *p* < 0.05.

## 3. Results

The detailed results of the repeated measures ANOVA for both the Flanker and Attentional Blink (AB) tasks are provided in Supplementary Tables S1 and S2. These tables present the main effects and interactions, along with effect sizes (ηp^2^) for all significant comparisons.

### 3.1. Correlation Matrix

Regarding the overall correlations for hits, the Flanker task shows a significant correlation with itself (r = 0.487), as does the Attentional Blink task (r = 0.533), as shown in Table 2. Additionally, for response times (see Table 3), the pretest scores of the Attentional Blink task correlate with the post-test scores of the Flanker task. The Flanker task indexes (pre and post) also correlate with themselves. However, this correlation is not observed between pre- and post-test values for the Attentional Blink task.

### 3.2. Flanker Task

#### 3.2.1. Response Times by Stimuli Type

Considering that mild, moderate, and even severe deviations from normality and homogeneity of variance do not compromise the robustness of the test—as it is capable of controlling Type I error within the limits of Bradley’s criterion [42]—the repeated measures ANOVA revealed significant differences in the variable Time (F(1,36) = 35.68, *p* < 0.001, ηp^2^ = 0.498), with a mean (M) of 693.61 and a standard deviation (SD) of 17.77 in the pretest and a mean of 618.72 and an SD of 13.61 in the post-test. However, there were no significant differences in the variable Type of stimuli (F(1,36) = 0.512, *p* = 0.479, ηp^2^ = 0.014), in the variable Group (F(2,36) = 0.160, *p* = 0.853, ηp^2^ = 0.009), or in their interaction (F(2,36) = 1.045, *p* = 0.362, ηp^2^ = 0.045; F(2,36) = 0.094, *p* = 0.911, ηp^2^ = 0.005).

#### 3.2.2. Interference Effect

Regarding the analysis of the interference effect on response times for congruent and incongruent stimuli, no significant differences were found for the variable Time (F(1,36) = 1.182, *p* = 0.284, ηp^2^ = 0.032), the variable Group (F(2,36) = 1.184, *p* = 0.318, ηp^2^ = 0.062), or their interaction (F(2,36) = 0.341, *p* = 0.713, ηp^2^ = 0.019). Therefore, there are no significant differences in interference effects between the groups.

#### 3.2.3. Correct Responses

Results from the 2 × 2 × 3 repeated measures ANOVA (Type × Time × Group) revealed a significant main effect of Time (F(1,36) = 8.280, *p* = 0.007, ηp^2^ = 0.187), indicating that participants were less accurate in the pretest (M = 22.34, SD = 0.28) compared to the post-test (M = 23.21, SD = 0.31). However, no significant differences were found for Type (F(1,36) = 1.016, *p* = 0.320) or Group (F(2,36) = 0.920, *p* = 0.408), and no significant interactions were observed (*p* > 0.050).

Since there was no effect of Type, an additional analysis was conducted to compare the total number of correct responses between the three groups in the pretest and post-test, regardless of congruence. This ANOVA showed a significant effect of Time (F(1,36) = 9.20, *p* = 0.004, ηp^2^ = 0.204), with more correct responses recorded in the post-test (M = 46.42, SD = 0.61) compared to the pretest (M = 44.61, SD = 0.57). However, no significant differences were found in the interaction between groups (F(1,36) = 1.604, *p* = 0.215, ηp^2^ = 0.082) or for the Group variable (F(2,36) = 0.893, *p* = 0.418, ηp^2^ = 0.047).

### 3.3. Attentional Blink Task

#### 3.3.1. Percentage of Hits

Although the Shapiro–Wilk analysis indicated that some comparisons deviated from normal distribution, the robustness of the test remained intact, provided that homogeneity of variance was maintained. This robustness allows the test to control Type I error rates within the boundaries set by Bradley’s criterion [43].

**Figure 3:**
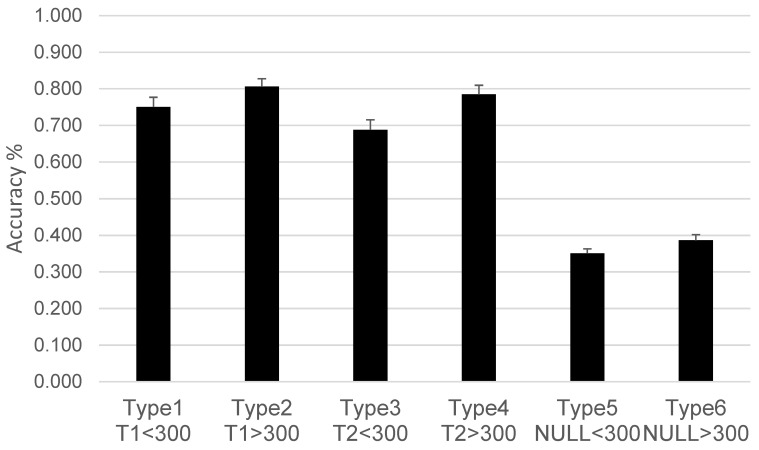
Accuracy by trial type in the Attentional Blink task. For trials type 1 to type 4, T1 refers to when the target stimulus appears in the first position, and T2 refers to when the target stimulus appears in the second position. The SOAs were either less than 300 ms or greater than 300 ms. Trials without target stimuli (type 5, type 6) showed significantly lower accuracy compared to the other types. Performance was also significantly worse in intervals <300 ms, where the Attentional Blink occurs. Mean ± SEM.

The first ANOVA in the Attentional Blink task revealed significant differences only for the variable Time (F(1,36) = 38.796, *p* < 0.001, ηp^2^ = 0.519). The second ANOVA revealed statistically significant differences according to the Type of trial (F(5,32) = 160.98, *p* < 0.001, ηp^2^ = 0.962) and Time (F(1,36) = 24.78, *p* < 0.001, ηp^2^ = 0.408), indicating that accuracy improved from the pretest (M = 0.58) to the post-test (M = 0.67), but not according to Group (F(2,36) = 2.75, *p* = 0.077). No statistically significant interactions were found. Post hoc analyses revealed several differences by Type (all *p* < 0.05), as shown in Figure 3. Type 5 and type 6 had significantly lower accuracy compared to type 1 through type 4 (all *p* < 0.001) but did not differ from each other (*p* = 0.051). Additional comparisons showed the following:

Type 1 had lower accuracy than Type 2 (*p* = 0.015).

Type 2 had higher accuracy than both Type 1 (*p* = 0.015) and Type 3 (*p* = 0.001).

Type 3 had lower accuracy than Type 2 (*p* = 0.001) and Type 4 (*p* < 0.001). The remaining comparisons were not significant (*p* > 0.05).

To further refine the analyses, trials were categorized based on the presence or absence of target stimuli. Accordingly, two additional repeated measures ANOVAs (Type × Time × Group) were conducted.

**Figure 4:**
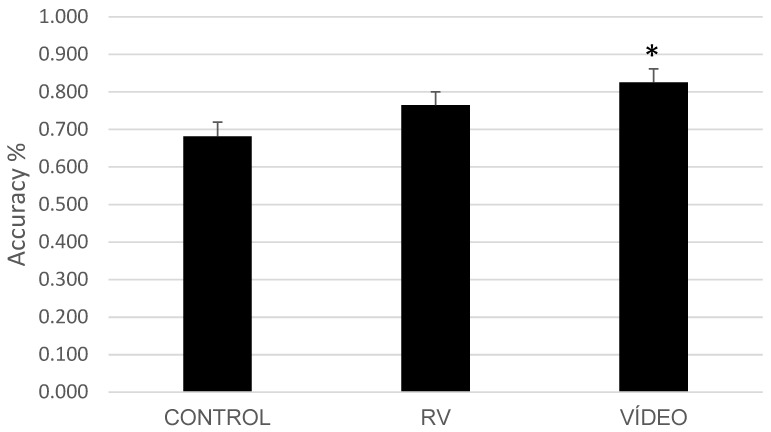
Accuracy by group for the Attentional Blink task. The VR video group demonstrated significantly higher accuracy than the Control group (* *p* < 0.05). Mean ± SEM.

For trials with target stimuli (types 1 to 4), there were significant differences for Type (F(3,34) = 13.76, *p* < 0.001, ηp^2^ = 0.549) and for Time (F(1,36) = 17.58, *p* < 0.001, ηp^2^ = 0.328). There was also a main effect of Group (F(2,36) = 3.74, *p* = 0.033, ηp^2^ = 0.172). No significant interactions were observed (*p* > 0.050). Post hoc analyses revealed that the VR video group (M = 0.82) had significantly higher accuracy compared to the control group (M = 0.68). However, the VR video group did not differ significantly from the RV group (M = 0.76), as illustrated in Figure 4.

Regardless of group, Type 1 had slower accuracy than Type 2 (*p* = 0.006). Type 3 had slower response times than both Type 2 (*p* < 0.001) and Type 4 (*p* < 0.001). The remaining comparisons were not significant (*p* > 0.05). Additionally, the pretest (M = 0.70, SD = 0.17) had lower accuracy scores compared to the post-test (M = 0.81, SD = 0.14).

For trials where the target was not present, there was a main effect of Type (F(1,36) = 9.84, *p* = 0.003, ηp^2^ = 0.215) and Time (F(1,36) = 23.97, *p* < 0.001, ηp^2^ = 0.400). The Group factor was not significant (F(2,36) = 0.124, *p* = 0.883). No significant interactions were observed (*p* > 0.05). Trials with SOAs more than 300 ms (M = 0.35, SD = 0.07) had lower accuracy compared to trials with SOAs less than 300 ms (M = 0.38, SD = 0.09). Independently, the pretest (M = 0.34, SD = 0.09) showed lower accuracy compared to the post-test (M = 0.39, SD = 0.08).

#### 3.3.2. Response Times

A repeated measures ANOVA was conducted to analyze response times for the Attentional Blink task, considering Stimulus Type (Present/Not Present) × Time (Pre/Post) × Group (Control, VR Exergame, VR Video). Significant differences were found for Stimulus Type (F(1,36) = 60.094, *p* < 0.001, ηp^2^ = 0.625), with present stimuli yielding a mean response time of M = 796.65 ms (SD = 32.1) and non-present stimuli yielding M = 925.51 ms (SD = 30.3). Significant differences were also found for Time (F(1,36) = 41.385, *p* < 0.001, ηp^2^ = 0.535), with longer response times in the pretest (M = 1035.40 ms, SD = 53.87) and shorter times in the post-test (M = 686.76 ms, SD = 19.42). In contrast, no significant differences were found between groups (F(2,36) = 1.755, *p* = 0.187, ηp^2^ = 0.089), nor were there significant interactions (F(2,36) = 2.687, *p* = 0.082, ηp^2^ = 0.13).

**Figure 5:**
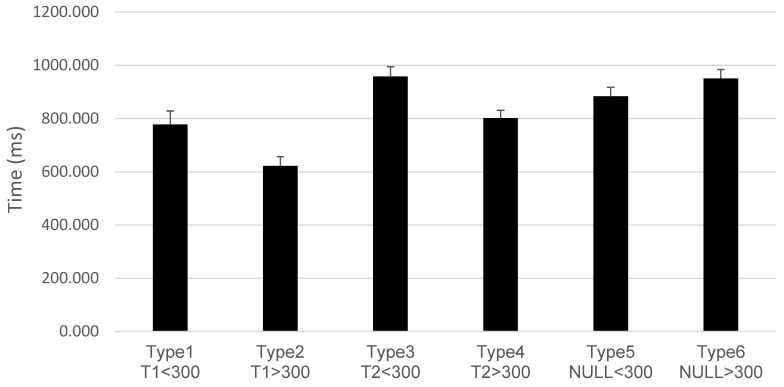
Response times by SOA in the Attentional Blink task. For the six types of trials, T1 refers to when the target stimulus appears in the first position, and T2 refers to when the target stimulus appears in the second position. The SOAs were either less than 300 ms or greater than 300 ms. Mean ± SEM.

A repeated measures ANOVA revealed a main effect for Type (F(5,32) = 26.94, *p* < 0.001, ηp^2^ = 0.808) and Time (F(1,36) = 39.93, *p* < 0.001, ηp^2^ = 0.526), but no significant effect for Group (F(2,36) = 1.43, *p* = 0.253). There were no significant interactions (*p* > 0.050). Post hoc analyses revealed several differences by Type (all *p* < 0.050), as shown in Figure 5:

Type 1: Slower than Type 2, but faster than Type 3, Type 5, and Type 6.

Type 2: Faster than all other types.

Type 3: Slower than Type 1, Type 2, and Type 4.

Type 4: Faster than Type 3, Type 5, and Type 6, but slower than Type 2.

Type 5: Slower than Type 2 and Type 4.

Type 6: Slower than Type 1, Type 2, and Type 4.

As for the Time, the pretest (M = 1013.31 ms, SD = 346.96) was slower than the post-test (M = 658.25, SD = 131.55).

As with the accuracy analysis, trials were divided based on the presence or absence of target stimuli. Two additional repeated measures ANOVAs (Type × Time × Group) were conducted.

For trials with target stimuli, there were significant differences for Type (F(3,34) = 41.503, *p* < 0.001, ηp^2^ = 0.786) and Time (F(1,36) = 39.95, *p* < 0.001, ηp^2^ = 0.526). The Group effect was not significant (F(2,36) = 1.42, *p* = 0.254). The interaction between Type and Group approached significance with Pillai’s Trace (F(6,70) = 2.19, *p* = 0.054, ηp^2^ = 0.158) and was significant with Wilks’ Lambda (F(6,68) = 2.30, *p* = 0.048, ηp^2^ = 0.166).

**Figure 6:**
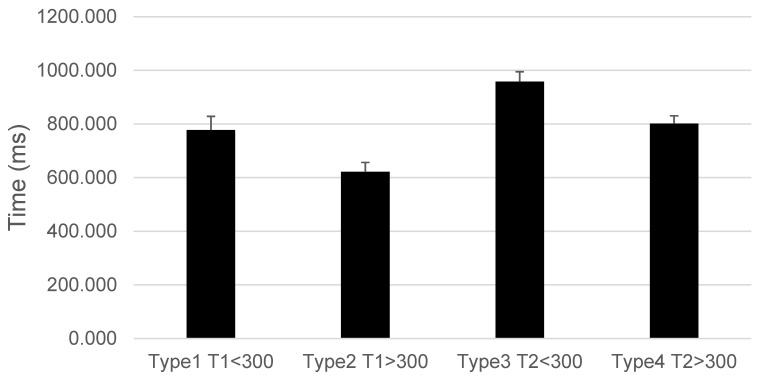
Response time by trial type in the Attentional Blink task. Significant differences were observed between types with SOAs < 300 ms, resulting in slower response times compared to SOAs > 300 ms. For the four types of trials, T1 refers to when the target stimulus appears in the first position, and T2 refers to when the target stimulus appears in the second position. The SOAs were either less than 300 ms or greater than 300 ms. Mean ± SEM.

Post hoc analyses for Time showed that the pretest (M = 975.72) was slower than the post-test (M = 604.09). Post hoc comparisons for Type indicated that Type 1 was faster than Type 3 (*p* < 0.001), Type 2 was faster than all other types (*p* < 0.001), and Type 3 was slower than Type 4 (*p* < 0.001). These results are illustrated in Figure 6.

**Figure 7:**
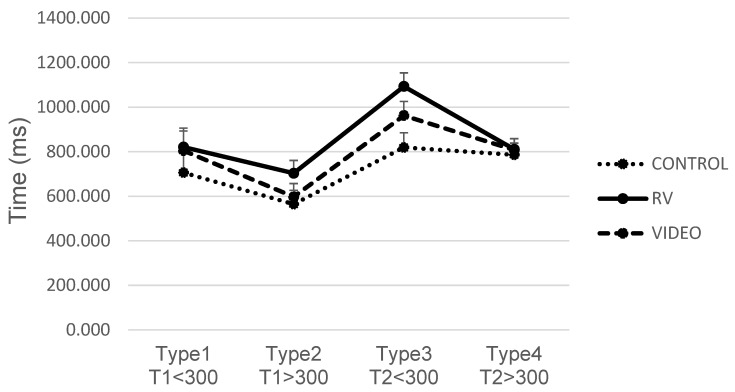
Analysis of the Type × Group interaction in the Attentional Blink task. For the four types of trials, T1 refers to when the target stimulus appears in the first position, and T2 refers to when the target stimulus appears in the second position. The SOAs were either less than 300 ms or greater than 300 ms. Mean ± SEM.

The interaction between Group and Type (see Figure 7) revealed that in inter-group comparisons for Type 3 (Target 2 in SOA < 300 ms trials), the VR exergame group (M = 1093.20; SD = 244.26) was slower than the Control group (M = 819.41, SD =134.04). Additionally, each group exhibited distinct patterns in intra-group Type comparisons:

- Control group: Type 2 was faster than both Type 3 and Type 4 (*p* < 0.001 for both).
- VR exergame group: Type 1 was faster than Type 3 (*p* = 0.001), and Type 2 was also faster than Type 3 (*p* < 0.001). Additionally, Type 3 was slower than Type 4 (*p* < 0.001).
- VR Video group: Type 1 was slower than Type 2 (*p* < 0.001). Type 2 was faster than all other types (*p* < 0.001). Type 3 was slower than both Type 2 (*p* < 0.001) and Type 4 (*p* = 0.036).

For non-target trials, there were significant differences according to Type (F(1,36) = 6.49, *p* = 0.015, ηp^2^ = 0.153) and Time (F(1,36) = 31.71, *p* < 0.001, ηp^2^ = 0.468). No significant differences were found according to Group (F(2,36) = 1.139, *p* = 0.331), and no interactions were significant (*p* > 0.050). Post hoc analyses revealed that trials with less than 300 ms between stimuli (M = 884.00) were slower than those with more than 300 ms between stimuli (M = 951.04). Additionally, the pretest (M = 1079.95) was slower than the post-test (M = 755.09).

## 4. Discussion

This study aimed to investigate the differential effects of a VR exergame, *Beat Saber*, and a nature-viewing experience, Ireland 4K, on attentional functions, as assessed by the Attentional Blink (AB) [34] and Flanker [15] tasks. The findings provide mixed support for our hypotheses, indicating that while both interventions have the potential to enhance cognitive functions, they do so in distinct ways.

The *Beat Saber* intervention, which combines mental and physical activity in a highly engaging environment, did not significantly enhance performance on the Flanker task, contrary to our expectations. This could be due to the specificity of the task, which primarily measures inhibitory control and selective attention, areas that might not be as strongly influenced by the general engagement and rapid response demands of *Beat Saber*.

In contrast, the Ireland 4K nature scenario, designed to provide a calming and restorative experience, significantly improved accuracy in the AB task, particularly in conditions with less time between stimuli (SOA < 300 ms). This supports the idea that nature exposure can enhance sustained attention and reduce cognitive fatigue, leading to better performance in tasks that require quick recovery of attentional resources. The lack of significant effects on Flanker task performance suggests that while nature viewing may enhance general attentional capacity, it does not necessarily improve selective attention or conflict resolution abilities in the same way as more active interventions might.

It is important to consider that the generalized improvements in response times and accuracy in the Flanker and Attentional Blink (AB) tasks in the post-assessment can be attributed to different factors. One possible factor that could explain these results is the training effect. Familiarization with the tasks through repetition can lead to the automation of responses and greater efficiency in stimulus processing, regardless of the intervention received [44]. This practice-related improvement is commonly observed in studies employing pre–post designs, where repeated exposure to the task leads to better performance in the second trial [45]. Additionally, the reduction in anxiety associated with increased familiarity with the experimental environment and task demands may have contributed to better performance in the post-test [46]. Initial anxiety, which may negatively impact performance during the first exposure to the tasks, could decrease in the second exposure, resulting in faster response times and greater accuracy. Another factor to consider is the potential adaptation to the experimental environment. Over time, participants may have adapted to the cognitive and contextual demands of the task, improving their concentration and processing efficiency during the second assessment [47]. This adaptation effect could explain the generalized improvement observed across all groups, beyond the specific intervention. Moreover, the inclusion of a rest period or a less demanding activity in the control and VR video groups may have allowed participants to recover from mental fatigue accumulated during the pretest, facilitating better performance in the post-test [48]. This recovery could explain why all groups, including the control group, showed improvements in performance after the intervention.

A deeper analysis of participants’ performance is warranted, given some particular effects observed in the cognitive tasks. The absence of interference effects, as indicated by the lack of significant differences between congruent and incongruent trials in the Flanker task, suggests that participants across all groups were equally capable of managing distractors, regardless of the intervention received. This finding could be interpreted in several ways. First, it is possible that the tasks used were not sufficiently demanding or did not generate enough cognitive load to elicit a typical interference effect, especially if participants were already adept at filtering out irrelevant information [15]. Additionally, the absence of an interference effect might suggest a ceiling effect, where participants across all groups had reached a level of performance that minimized the impact of distractors, possibly due to the familiarity with the task format acquired during the pretest phase and their previous experience in similar experiments as university psychology students [49]. Furthermore, age and good physical condition have been shown to be key factors that significantly influence cognitive task performance, especially in selective and sustained attention [50], and our sample was limited to young university students, who tend to show optimal cognitive training.

Another explanation could involve the specificity of the interventions used. While VR exergames and nature video viewing are designed to enhance attentional control and cognitive flexibility, they may not directly influence the selective attention processes required to differentiate between congruent and incongruent stimuli in the Flanker task [51]. The generalized improvement in response times observed across all trial types could reflect overall enhancements in processing speed and motor responses rather than specific gains in inhibitory control. This suggests that while the interventions might improve general cognitive functioning, they do not differentially impact the ability to manage interference from distractors in the Flanker task. Future research could consider using more complex or variable stimuli to better capture potential differences in selective attention capabilities [52].

The Attentional Blink (AB) task results mirrored those observed in the Flanker task, with no significant differences between the intervention groups. However, significant differences emerged when examining the type of trial, particularly between trials without target stimuli (Types 5 and 6) and those with target stimuli (Types 1 through 4).

The significantly lower accuracy and longer response time in trials without target stimuli (Type 5: no stimulus present, SOA < 300 ms, and Type 6: no stimulus present, SOA > 300 ms) compared to trials where a target stimulus was present (Types 1–4) indicate that the absence of targets led to increased difficulty in maintaining attentional focus or detecting the absence of stimuli. This pattern is consistent with the notion that participants may have found it more challenging to sustain attention or correctly identify the absence of a target when no stimulus was presented, leading to more errors [37]. The lack of significant differences between Type 5 and Type 6 trials further suggests that the length of the SOA alone may not have been sufficient to influence accuracy when no targets were present. However, trials with SOA < 300ms were slower, indicating that the temporal proximity of potential targets did affect processing speed. This points to a nuanced effect of SOA on task performance, where the timing of potential stimuli primarily affected response speed rather than the accuracy of detecting their absence.

When considering trials with target stimuli (Types 1–4), the effect of SOA was evident in both accuracy and response time. Type 1 trials (Target 1, SOA < 300 ms) had lower accuracy than Type 2 trials (Target 1, SOA > 300 ms), indicating that shorter intervals between stimuli led to greater difficulty in accurately detecting the first target. This result aligns with the classic understanding of the Attentional Blink, where a short SOA can overwhelm the attentional system, reducing the ability to process and accurately identify stimuli [16].

Response time data further supports these findings. Post hoc analyses revealed that Type 1 (Target 1, SOA < 300 ms) trials were faster than Type 3 (Target 2, SOA < 300 ms), and Type 2 (Target 1, SOA > 300 ms) trials were faster than all other types. This suggests that participants were able to respond more quickly when the first target appeared, particularly when the SOA was longer, allowing the attentional system more time to process the first stimulus. The slower response times observed in Type 3 (Target 2, SOA < 300 ms) compared to Type 4 (Target 2, SOA > 300 ms) further indicate that when the second target appeared shortly after the first (SOA < 300 ms), participants experienced a greater delay in response, likely due to the increased cognitive load associated with processing two targets in quick succession.

The comparison between Target 1 and Target 2 trials highlights the impact of SOA on both accuracy and response time. While Type 1 (T1, SOA < 300 ms) did not differ significantly from Type 3 (T2, SOA < 300 ms) in accuracy, the response times suggest that the attentional system was particularly taxed when processing the second target quickly after the first. The reduced accuracy and slower response times in these conditions reflect the attentional system’s difficulty in quickly resetting and refocusing on the second target after processing the first [53].

The analysis of trials with target stimuli (Types 1 to 4) revealed a main effect of Group, suggesting that the type of intervention influenced accuracy, with the VR video group showing higher accuracy compared to the control group. However, the lack of significant differences in the post hoc analysis between the VR video and VR exergame groups suggests that while the VR video intervention may have had a beneficial impact on attentional performance, the effect was not robust enough to produce clear distinctions among the intervention groups. This could indicate that the observed differences might be due to general engagement or the calming effects of nature scenes rather than a specific enhancement of attentional control [13,51]. In contrast, the analysis of response times for these same trials did not show a significant main effect for Group. However, the interaction between Type and Group approached significance with Pillai’s Trace and was significant with Wilks’ Lambda, indicating that the effect of the intervention varied depending on the specific trial type. Notably, the interaction between the Group and Type of the trial revealed that, in inter-group comparisons for Type 3 (Target 2 in SOA < 300 ms trials), the VR exergame group was slower than the control group. This suggests that while the VR video group demonstrated overall higher accuracy, the VR exergame group struggled more with processing speed in conditions where the second target appeared shortly after the first.

Overall, the results of the Flanker and AB tasks demonstrate that while no significant differences were observed between the intervention groups, the manipulation of SOA and the presence or absence of target stimuli significantly influenced task performance. These findings underscore the robustness of the Attentional Blink phenomenon and the challenges inherent in processing rapidly presented sequential stimuli, particularly when attentional resources are taxed by closely spaced targets. However, these results contrast with previous studies that suggest playing video games in virtual reality tends to improve certain cognitive abilities, specifically attention [54].

One potential explanation for this discrepancy is the duration of the intervention. Our study utilized a brief 20 min intervention, which may not have been sufficient to generate significant changes in attentional abilities. This is consistent with the literature suggesting that attentional effects typically manifest after longer or more prolonged training sessions. For instance, Perea-Lozano and Peña-Álvarez [18] found that experienced video game players showed improvements in selective attention, cognitive flexibility, and reduced interference levels, likely due to their extensive gaming experience. Similarly, Flores-Gallegos et al. [22] demonstrated that a regimen of 30 sessions, each lasting 30 min, including *Beat Saber* and GrafoTami, led to a significant decrease in post-test error rates.

Furthermore, previous research on the effects of nature exposure on attention has yielded mixed results. Hartig et al. [55] showed that depending on the specific attentional task used, attention may be enhanced after exposure to natural environments (e.g., Necker Cube Pattern Control Test—NCPCT) or may not show any improvement (e.g., Search Memory Test—SMT). Hartig et al. [56] attributed the lack of effect in the SMT task to the brief duration of exposure (50 min). Additionally, Hartig et al. [55] noted that neither natural nor urban stimuli produced effects in the SMT task. Ohly et al. [57] further highlighted the heterogeneity of results in their meta-analysis, noting that only the most demanding attentional tasks (e.g., Digit Span Forward, Digit Span Backward, and Trail Making Test B) consistently demonstrated the restorative effects of nature on attention. Interestingly, contrary to studies supporting the positive influence of nature exposure, Ohly et al. [57] found that tasks like the SMT yielded better results after exposure to an urban environment. They suggested that these effects may be more evident in tasks measuring inhibition capacity rather than attention, although our study did not find such effects in the Flanker task.

The attentional tasks included in Ohly et al. [57] meta-analysis have a significant working memory component, unlike the Flanker task and the AB task used in our study. Our objective in selecting these two tasks was to minimize the influence of other cognitive processes, such as working memory, and focus specifically on attentional control [58,59,60]. We observed that inhibitory control and working memory are separate processes, and while rhythm training can improve working memory in tasks like the Corsi test, it does not necessarily enhance inhibitory control in the Flanker task.

Our study aimed to test the acute effect of a single session of either playing a VR exergame or being exposed to a VR video displaying nature scenes, based on previous studies suggesting that exergames can mimic the benefits of moderate exercise in improving inhibitory responses [61]. Additionally, Sahni and Kumar [62] found that watching a 15 min video with nature scenes produced improvements in inhibitory control processes as measured by the Flanker task. However, some research has shown that acute exercise yields cognitive improvements, while exergaming does not [63]. And recent research by Hartanto et al. [64] also did not find evidence that brief exposure to virtual nature, such as watching 15 min videos of natural scenery, significantly restores attentional resources. This suggests that virtual nature simulations, even high-quality ones, may not replicate the restorative effects of direct outdoor experiences. The sensory richness, physical presence, and multisensory stimuli in real nature are likely critical for attentional restoration, and these are challenging to fully simulate in virtual environments.

### Limitations and Future Research Directions

Although the present study did not show significant improvements in attentional performance following brief VR exposure, the findings underscore the challenges posed by rapid attentional shifts, as demonstrated by the Attentional Blink (AB) phenomenon. The persistence of the AB effect in this study suggests that VR training interventions may need to be longer or more tailored to address these specific attentional limitations. Future VR training programs could benefit from incorporating extended or repeated sessions, progressively challenging users with closely spaced stimuli to train and potentially reduce the blink effect. The immersive and interactive nature of VR provides an opportunity to design adaptive training environments that mimic real-world multitasking scenarios, gradually reducing the SOA between stimuli as users improve. Additionally, the use of more challenging or complex tasks that engage multiple cognitive processes, such as working memory or executive function, may reveal differential effects of the interventions that were not apparent in our study.

Another limitation is the sample composition. Given that participants were psychology students who are often exposed to similar experimental paradigms, their familiarity with the tasks might have influenced the results. Additionally, to detect small or subtle effects on attentional function, it is likely that a larger sample size will be required. Future studies should include a more diverse sample to generalize the findings and control for prior exposure to cognitive tasks.

Lastly, it would be beneficial to examine the long-term effects of these interventions on cognitive performance and to explore whether any observed benefits persist or accumulate over time. Incorporating neuroimaging techniques could also provide insights into the neural mechanisms underlying any cognitive improvements resulting from VR-based interventions.

## 5. Conclusions

This study explored the effects of a single session of a VR exergame (*Beat Saber*) and a VR nature video on attentional performance using the Flanker and Attentional Blink (AB) tasks. Our findings revealed significant improvements in response times and accuracy across all groups in post-test measures, suggesting a strong training effect rather than a specific impact from the interventions.

Significant effects were observed in the AB task, where the manipulation of stimulus onset asynchrony (SOA) and the presence or absence of target stimuli played a crucial role. Shorter SOAs led to decreased accuracy and slower response times, emphasizing the difficulty of processing closely spaced targets. Additionally, the interaction between Type and Group in response times for target stimuli indicated that the intervention types differentially influenced processing speed under certain conditions.

Overall, while the brief interventions did not produce distinct differences between groups, the results underscore the influence of task-specific factors, such as SOA and target presence, on performance. Future VR interventions could build on these insights by incorporating progressively challenging tasks, adjusting stimulus intervals to train rapid attention shifts, and engaging multiple cognitive processes like working memory and executive function. These applications hold promise for educational and therapeutic settings, where enhancing attentional control and cognitive flexibility is essential for learning and rehabilitation. Even brief sessions of VR-based training show potential for scalability, offering a valuable tool for cognitive development in diverse populations and settings. Further research is needed to explore the long-term benefits of these immersive interventions and refine their application for maximum cognitive gains.

## Figures and Tables

**Table 1:** Sequence of congruent and incongruent stimuli.

Congruent	Incongruent
XXXXX	XXBXX
CCXCC	XXVXX
XXCXX	CCVCC
CCCCC	CCBCC
BBBBB	VVCVV
VVVVV	BBCBB
VVBVV	VVXVV
BBVBB	BBXBB

**Table 2:** Correlation matrix (r values) for hits across tasks.

	AB. PRE	AB. POST	FLA. PRE	FLA. POST
AB. PRE	1	0.533 **	0.247	0.122
AB.POST		1	0.130	0.458
Flanker PRE			1	0.487 **
Flanker POST				1

** *p* < 0.01.

**Table 3:** Correlation matrix (r values) for response times across tasks.

	AB. PRE	AB. POST	FLA. PRE	FLA. POST
AB. PRE	1	0.090	−0.156	−0.346 *
AB.POST		1	−0.75	−0.088
Flanker PRE			1	0.712 **
Flanker POST				1

** *p* < 0.01; * *p* < 0.05.

## Data Availability

Dataset available at https://figshare.com/s/7db5fd674c966d807372 (accessed on 22 September 2024).

## Supplementary Materials

The following supporting information can be downloaded at: https://www.mdpi.com/article/10.3390/brainsci14100972/s1, Table S1: Summary of repeated measures ANOVA results for the Flanker Task. Effect sizes reported for significant comparisons; Table S2: Summary of repeated measures ANOVA (Type × Time × Group) for the Attentional Blink Task. Effect sizes reported for significant comparisons.
