# Supplementary material for: Comparison of Virtual Reality Exergames and Nature Videos on Attentional Performance: A Single-Session Study
Source: Brain Sci. 2024 Sep 26;14(10):972. doi: 10.3390/brainsci14100972 (PMC11506412; doi:10.3390/brainsci14100972)
Supplement: Supplementary file 1 [file brainsci-14-00972-s001.zip › brainsci-3207883-supplementary.pdf]

Table S1. Summary of repeated measures ANOVA results for the Flanker Task. Effect sizes reported for significant comparisons.

**Response Times**				
Effect	F Degrees offreedom	F Value	P Value	Effect Size (ηp^2)
Time	(1,36)	35.68	<0.001*	0.498
Type	(1,36)	0.512	0.479	-
Group	(2,36)	0.160	0.853	-
**Interference Effect**				
Effect	F Degrees offreedom	F Value	P Value	Effect Size (ηp^2)
Time	(1,36)	1.182	0.284	-
Group	(2,36)	1.184	0.318	-
**Correct Responses (Type x Time x Group)**				
Effect	F Degrees offreedom	F Value	P Value	Effect Size (ηp^2)
Time	(1,36)	8.280	0.007	0.187
Type	(1,36)	1.016	0.320	-
Group	(2,36)	0.920	0.408	-
**Correct Responses (Time x Group)**				
Time	(1,36)	9.20	0.004*	0.204
Group	(2,36)	1.604	0.215	-

*$p < 0.05$.

Table S2. Summary of repeated measures ANOVA (Type x Time x Group) for the Attentional Blink Task. Effect sizes reported for significant comparisons.

Effect	F Degrees of freedom	F Value	P Value	Effect Size (ηp^2)
**Percentage of hits (all trials)**				
Time	(1,36)	24.78	<0.001*	0.408
Type	(5,32)	160.98	<0.001*	0.962
Group	(2,36)	2.75	0.077	-
**Percentage of hits (Present)**				
Effect	F Degrees of freedom	F Value	P Value	Effect Size (ηp^2)
Time	(1,36)	17.58	<0.001*	
Type	(3,34)	13.76	<0.001*	0.549
Group	(2,36)	3.74	0.033*	0.172
**Percentage of hits (Absence)**				
Effect	F Degrees of freedom	F Value	P Value	Effect Size (ηp^2)
Type	(1,36)	9.84	0.003*	0.215
Time	(1,36)	23.97	<0.001*	0.400
Group	(2,36)	0.124	0.883	
**Response Times (all trials)**				
Effect	F Degrees of freedom	F Value	P Value	Effect Size (ηp^2)
Time	(1,36)	41.385	0.001*	0.535
Type	(1,36)	60.094	0.001*	0.625
Group	(2,36)	1.755	0.187	-
**Response Times (Present)**				
Effect	F Degrees offreedom	F Value	P Value	Effect Size (ηp^2)

Time	(1,36)	39.95	<0.001*	0.526
Type	(3,34)	41.503	<0.001*	0.786
Group	(2,36)	1.42	0.254	-
**Response Times (Absence)**				
Effect	F Degrees offreedom	F Value	P Value	Effect Size (ηp^2)
Time	(1,36)	31.71	<0.001*	0.468
Type	(1,36)	6.49	<0.015*	0.786
Group	(2,36)	1.139	0.331	-

*$p < 0.05$.
